# Effects of oestradiol and tamoxifen on VEGF, soluble VEGFR-1, and VEGFR-2 in breast cancer and endothelial cells

**DOI:** 10.1038/sj.bjc.6602824

**Published:** 2005-10-18

**Authors:** S Garvin, U W Nilsson, C Dabrosin

**Affiliations:** 1 Division of Gynecologic Oncology, University Hospital, SE-581 85 Linköping, Sweden

**Keywords:** breast cancer, flt-1, flk-1, KDR, MCF-7, nude mice

## Abstract

Angiogenesis is regulated by the balance between pro- and antiangiogenic factors. Vascular endothelial growth factor (VEGF), acting via the receptors VEGFR-1 and VEGFR-2, is a key mediator of tumour angiogenesis. The soluble form of the VEGF receptor-1 (sVEGFR-1) is an important negative regulator of VEGF-mediated angiogenesis. The majority of breast cancers are oestrogen dependent, but it is not fully understood how oestrogen and the antioestrogen, tamoxifen, affect the balance of angiogenic factors. Angiogenesis is a result of the interplay between cancer and endothelial cells, and sex steroids may exert effects on both cell types. In this study we show that oestradiol decreased secreted sVEGFR-1, increased secreted VEGF, and decreased the ratio of sVEGFR-1/VEGF in MCF-7 human breast cancer cells. The addition of tamoxifen opposed these effects. Moreover, human umbilical vein endothelial cells (HUVEC) incubated with supernatants from oestradiol-treated MCF-7 cells exhibited higher VEGFR-2 levels than controls. *In vivo*, MCF-7 tumours from oestradiol+tamoxifen-treated nude mice exhibited decreased tumour vasculature. Our results suggest that tamoxifen and oestradiol exert dual effects on the angiogenic environment in breast cancer by regulating cancer cell-secreted angiogenic ligands such as VEGF and sVEGFR-1 and by affecting VEGFR-2 expression of endothelial cells.

Vascular endothelial growth factor (VEGF) is a key mediator of tumour angiogenesis, including neovascularisation in human breast cancer (Folkman, 1995). Vascular endothelial growth factor is known to exert its angiogenic effects via two tyrosine kinase receptors, VEGFR-1 (flt-1) and VEGFR-2 (flk-1/KDR) (Ferrara, 1999; Neufeld *et al*, 1999). Knockout experiments have demonstrated that both VEGFR-1 and VEGFR-2 are crucial for normal development of the embryonic vasculature (Fong *et al*, 1995; Shalaby *et al*, 1995). Whereas VEGFR-2 appears to mediate differentiation, migration, and proliferation of endothelial cells, VEGFR-1 seems to regulate maintenance of blood vessels in later developmental stages (Hanahan, 1997). In addition, a naturally occurring soluble form of VEGFR-1 has been identified (Shibuya *et al*, 1990; Kendall and Thomas, 1993). This alternatively spliced variant of VEGFR-1 with a unique C-terminal extension of 31 residues binds VEGF with high affinity (Kendall and Thomas, 1993). Recombinant sVEGFR-1 has been found to bind all VEGF isoforms and to inhibit endothelial cell proliferation induced by VEGF (Kendall *et al*, 1996). Although the exact physiological role and mechanism of this receptor is yet unknown, it is thought that sVEGFR-1 exerts biological activity in the extracellular space, possibly by sequestering VEGF and limiting its bioavailability (Hornig and Weich, 1999).

It has been suggested that sVEGFR-1 may be an important negative regulator of VEGF in breast cancer (Toi *et al*, 2002). Breast cancer patients have been found to have significantly higher plasma levels of VEGF and significantly lower plasma levels of sVEGFR-1 compared to healthy controls (Caine *et al*, 2003). Moreover, the expression of sVEGFR-1 has been demonstrated in breast tumour tissues, and it has been described that the ratio of sVEGFR-1 to VEGF is a strong prognostic indicator of disease-free and overall survival in breast cancer patients (Toi *et al*, 2002; Bando *et al*, 2005).

The predominant localisation of VEGFR-2 has been shown by immunohistochemistry-based analysis to be in endothelial cells, yet expression has also been identified in epithelial cells in breast cancer (de Jong *et al*, 1998; Kranz *et al*, 1999; Ryden *et al*, 2003). In addition, it has been shown that the expression of activated VEGFR-2 is higher in malignant breast tissue as compared to neighbouring non-neoplastic regions (Kranz *et al*, 1999).

The majority of primary breast tumours are oestrogen-dependent, and tamoxifen is the most commonly used antioestrogen therapy today. Numerous animal models of breast cancer have previously demonstrated a proangiogenic effect of oestrogens and an antiangiogenic effect of tamoxifen *in vivo* (Haran *et al*, 1994; Lindner and Borden, 1997; Guo *et al*, 2002; Dabrosin *et al*, 2003a, 2003b; Garvin and Dabrosin, 2003; Elkin *et al*, 2004). We have previously shown that oestradiol increases extracellular levels of VEGF while tamoxifen inhibits the secretion of VEGF in breast cancer *in vivo* (Garvin and Dabrosin, 2003). However, very little is known about the effects of oestradiol and tamoxifen on VEGF receptors and the angiogenic microenvironment in breast cancer. In this study, we have investigated the effects of oestradiol and tamoxifen on sVEGFR-1 and VEGFR-2 in human cell lines *in vitro* and a mouse model of breast cancer *in vivo*. In addition, we have explored the effects of oestradiol and tamoxifen on endothelial cell expression of VEGF and VEGFR-2.

## MATERIALS AND METHODS

### Cells and culture conditions

MCF-7 cells (ER+ and PR+; ATCC, Manassas, VA, USA) were cultured in Dulbecco's modified Eagle's medium without phenol red supplemented with 2 mM glutamine, 50 IU ml^−1^ penicillin-G, 50 *μ*g ml^−1^ streptomycin, and 10% foetal bovine serum (FBS) at 37°C in a humidified atmosphere containing 5% CO_2_. Cell culture media and additives were obtained from GIBCO, Paisley, UK if not otherwise stated. Prior to experiments, cells were trypsinised (0.05% trypsin and 0.02% EDTA) and seeded into Petri dishes (Costar, Cambridge, MA, USA), 10 000 cells cm^−2^. Cells were incubated for 1 day and thereafter treated with or without 10^−8^ M oestradiol (17*β*-oestradiol; Apoteket, Umeå, Sweden), 10^−6^ M tamoxifen (Sigma, St Louis, MO, USA) or a combination of oestradiol and tamoxifen. The dose of oestradiol may be considered physiological, taking into consideration the local production and accumulation of oestradiol in human breast tumours *in vivo* (van Landeghem *et al*, 1985; Vermeulen *et al*, 1986; Pasqualini *et al*, 1996). The concentration of tamoxifen is equivalent to therapeutic serum concentrations found in breast cancer patients. Hormones were added to the MCF-7 cultures in serum-free medium consisting of a 1 : 1 mixture of nutrient mixture F-12 (HAM) and Dulbecco's modified Eagle's medium without phenol red supplemented with 10 *μ*g ml^−1^ transferrin (Sigma), 1 *μ*g ml^−1^ insulin (Sigma), and 0.2 mg ml^−1^ bovine serum albumin (Sigma). The medium was changed every day. Secreted VEGF and sVEGFR-1 was quantified in medium collected from day 7.

Umbilical cords were donated anonymously after informed consent according to national ethical legislation. Human umbilical vein endothelial cells (HUVEC) were isolated from freshly delivered female donor umbilical veins by collegenase digestion at 37°C for 15 min according to established methods (Jaffe *et al*, 1973). Cells were grown in medium consisting of Dulbecco's modified Eagle's medium without phenol red supplemented with nonessential amino acids, 1.6 mM glutamine, 4 IU ml^−1^ penicillin-G, 4 *μ*g ml^−1^ streptomycin, 4 *μ*g ml^−1^ insulin, 0.01 M HEPES, 0.02 mg ml^−1^ endothelial cell growth factor (ECGF; Roche Diagnostics, Bromma, Sweden), 16 IE ml^−1^ heparin (Apoteket, Umeå, Sweden), and 16% FBS and incubated at 37°C in a humidified atmosphere containing 5% CO_2_. Cells used for experiments were from passages 2 to 3. In order to eliminate the oestrogen-like activity present in FBS, the cells were switched 24 h before the start of hormone treatment to growth medium as described above but containing 16% charcoal-filtered FBS instead of nonfiltered FBS. For determination of VEGF and VEGFR-2 cells were trypsinised (0.05% trypsin and 0.02% EDTA) and seeded into Petri dishes precoated with 0.2% gelatin (Costar, Cambridge, MA, USA), 20 000 cells cm^−2^. Cells were treated with or without 10^−8^ M oestradiol (17*β*-oestradiol; Apoteket, Umeå, Sweden), 10^−6^ M tamoxifen (Sigma, St Louis, MO, USA) or a combination of oestradiol and tamoxifen. The medium was changed every day. At 3 days was chosen for the duration of treatment with MCF-7 supernatants due to the shorter survival time of HUVEC without the medium supplements described above. Secreted VEGF was quantified in medium collected from day 3. For experiments with conditioned medium, MCF-7 cell supernatants from oestradiol, tamoxifen, and control groups collected from days 1, 2, and 3 were added to HUVEC daily. Supernatants from MCF-7 cells were collected from dishes with equivalent cell counts in all treatment groups.

### Quantification of VEGF, sVEGFR-1, and VEGFR-2

Cell culture media and cell lysates were analyzed for VEGF, sVEGFR-1, and VEGFR-2 using commercial quantitative immunoassay kits for human VEGF (QuantGlo, human VEGF; R&D systems, Abingdon, UK), human sVEGFR-1 (Quantikine, human sVEGFR-1; R&D systems, Abingdon, UK), or human VEGFR-2 (Quantikine, human VEGFR-2; R&D systems, Abingdon, UK) without preparation. Following the removal of cell medium, the cells were washed with PBS, frozen in −20°C and thawed three times, diluted in PBS, and sonicated for 10 s. Protein content was determined using Bio-Rad DC Protein Assay (Bio-Rad, Sundbyberg, Sweden). According to the manufacturer, the VEGF kit measures the VEGF 165 and 121 isoforms and the minimum detectable dose is less than 1.76 pg ml^−1^, intra-assay and inter-assay precision 3–8%. The minimum detectable dose is 5.01 pg ml^−1^ for the sVEGFR-1 kit and 4.6 pg ml^−1^ for the VEGFR-2 kit according to the manufacturer. The intra-assay and inter-assay precision for these kits are 3–8 and 3–7%, respectively. All three kits are designed to eliminate interference by soluble receptors or ligands and other binding proteins. The sVEGFR-1 kit detects both VEGFR-1 and sVEGFR-1. For all experiments *n* equals the total number of dishes analyzed per treatment arm from repeated experiments as specified in the figure legends.

### Animals and oophorectomy of mice

Female athymic mice (6–8 weeks old) were purchased from M&B, Denmark. They were housed in a pathogen-free isolation facility with a light/dark cycle of 12/12 h and fed with rodent chow and water *ad libitum*. The Linköping University animal ethics research board approved all animal work, and the *in vivo* procedures used were fully consistent with the animal use guidelines of the UKCCCR (Workman *et al*, 1998). Mice were anesthetised with i.p. injections of ketamine/xylazine, oophorectomised (OVX), and 3-mm pellets containing 17 *β*-oestradiol, 0.18 mg 60-d^−1^ release, or placebo pellets (Innovative Research of America, Sarasota, Florida, USA) were implanted subcutaneously in the animal's back 7 days before tumour induction. The pellets provide continuous release of oestradiol at serum concentrations of 150–250 pM, confirmed previously by serum analysis(Dabrosin *et al*, 2003b), which is in the range of physiologic levels during the estrous cycle in mice. At 1 week after surgery, MCF-7 cells (5 × 10^6^ cells in 200 *μ*l PBS) were injected s.c. on the right hind side flank. Tumour volume was determined by measuring length, width, and depth of the tumour every 5 days using a caliper. At a tumour size of approximately 300 mm^3^ (approximately 6–7 weeks after tumour-cell injection), the mice were divided into two subgroups, *n*=5–8 in each group. One group continued with the oestradiol treatment only, while tamoxifen (1 mg/every two days s.c.) was added to the oestradiol treatment for 2 weeks in the other group. MCF-7 tumours are dependent on oestradiol for growth in nude mice (Shafie, 1980). Therefore, it was not possible to include an untreated control group or a tamoxifen-alone group in the experimental design.

### Immunohistochemistry of tumour sections

Formalin-fixed, paraffin embedded tumours were sectioned, deparaffinised and subjected to anti-human VEGFR-1 immunohistochemistry (goat anti-human VEGFR-1, dilution 1 : 10, R&D systems) or anti-von Willebrand's factor (rabbit anti-human von Willebrand; dilution 1 : 1000, DakoCytomation). The anti-human VEGFR-1 antibody is specific for the extracellular domain of rhVEGFR-1 and detects both VEGFR-1 and sVEGFR-1. Sections were counterstained with Mayer's hematoxylin. Negative controls did not show staining. VEGFR-1 scoring was conducted in a blinded manner. The whole material, all sections, was first scanned to determine the range of intensity of the staining. The intensity of VEGFR-1 staining in each section was thereafter scored as weakly or strongly positive in 10 high power fields (× 200) examined in sections from three different tumours in each group. Vessel quantification of tumour sections was performed as previously described using a Nikon microscope equipped with a digital camera (Schor *et al*, 1998). In a blinded manner, the section was first scanned and three hot-spots were selected for vessel area quantification (× 200). Three different tumours in each group were measured. The percentage of area with positive staining for von Willebrand's factor was assessed using Easy Image Measurement software (Bergstrom Instruments). Tumour sections were also subjected to H&E staining.

### Statistics

The values represent the mean ± SEM. Statistical analyses were performed with Student's *t*-test, ANOVA with Fisher's *post hoc* test, and Fisher's exact test where appropriate.

## RESULTS

**Figure 1:**
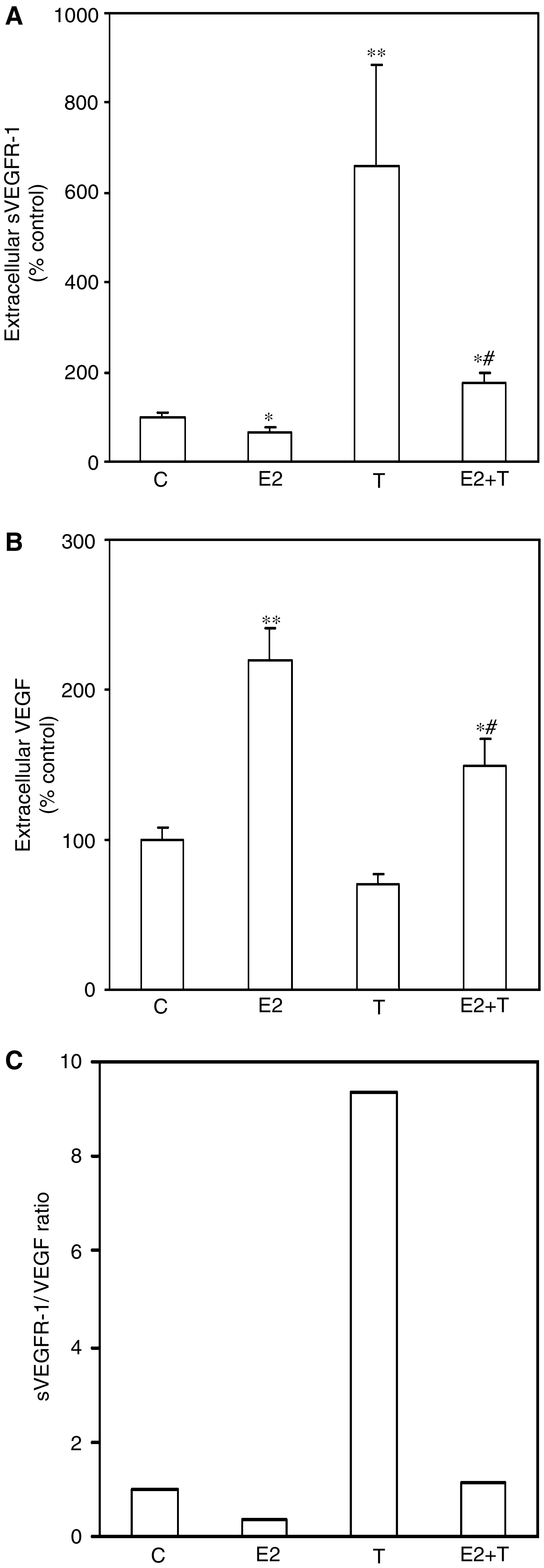
Extracellular sVEGFR-1, VEGF, and sVEGFR-1/VEGF ratio after hormone exposure of MCF-7 cells in culture. MCF-7 cells were cultured without hormones (C) or in the presence of oestradiol (E2; 10^−8^ M), tamoxifen (T; 10^−6^ M), or a combination of E2+T for 7 days. (**A**) sVEGFR-1 in cell culture media measured using ELISA (*n*=6–8 in each group, four separate experiments; ^**^P<0.01, ^*^P<0.05 as compared with control cells, #*P*<0.01 as compared with oestradiol-treated cells). (**B**) Extracellular VEGF measured using ELISA (*n*=4–5 in each group, two separate experiments; ^**^P<0.01, ^*^P<0.05 as compared with control cells, #*P*<0.05 as compared with oestradiol-treated cells). (**C**) sVEGFR-1/VEGF ratio based on extracellular levels of sVEGFR-1 and VEGF measured using ELISA and expressed as percent of control.

After 7 days of hormone exposure, a significantly higher level of secreted sVEGFR-1 was detected in MCF-7 cells treated with tamoxifen as compared to control cells (*P*<0.01; Figure 1A) whereas oestradiol-treatment decreased sVEGFR-1 levels (*P*<0.05 as compared to control cells; Figure 1A). In line with our previous studies (Garvin and Dabrosin, 2003), the combination of tamoxifen and oestradiol significantly decreased the secretion of VEGF (*P*<0.05 as compared to oestradiol-treated cells) while oestradiol significantly increased secreted VEGF (*P*<0.01 as compared to controls; Figure 1B). Extracellular levels of VEGF in pg mg^−1^ protein ranged from approximately 500 pg mg^−1^ protein in tamoxifen-treated cell medium to 1500 pg mg^−1^ protein in oestradiol-treated cell medium.

Likewise, levels of cell-associated sVEGFR-1/VEGFR-1 were significantly higher in tamoxifen-treated MCF-7 cells and significantly lower in oestradiol-treated cells; cell-associated sVEGFR-1/VEGFR-1 levels were 840±300% of control in the tamoxifen group (*P*<0.01 compared to control cells, 100±5%), 69±9% in the oestradiol group (*P*<0.05 compared to control cells), and 150±27% in the oestradiol+tamoxifen group. In the light of a recent report that different types of lysis buffers affect measurements of sVEGFR-1 (Bando *et al*, 2005), it may be noted that no lysis buffer was used in this experiment and all treatment groups were prepared in the same manner.

The ratio between sVEGFR-1 and VEGF (in percent of control) was calculated in order to gain better insight into the biological relevance of sex steroid effects on the angiogenic environment. The extracellular sVEGFR-1/VEGF ratio in MCF-7 cell medium following 7 days of tamoxifen treatment was approximately nine times greater than that of control cells, while the ratio in oestradiol-treated cells was nearly one-third that of controls. The combination of oestradiol and tamoxifen yielded a ratio approximately equivalent to that of control cells (Figure 1C). This pattern was also observed in cell lysates, although to a slightly lesser extent, with a five times greater sVEGFR-1/VEGF ratio in tamoxifen-treated cells as compared to controls after 7 days treatment. The ratio in oestradiol-treated cells was again approximately one-third of that of controls. It has been suggested that each subunit of dimeric VEGF binds one molecule of sVEGFR-1 (Kendall and Thomas, 1993). Calculating the molar sVEGFR-1/VEGF ratio based on nmol mg^−1^ protein revealed a similar pattern, with a proangiogenic effect on the ratio by oestradiol and an antiangiogenic effect by tamoxifen; the ratio in oestradiol-treated cells was approximately one half of that of controls compared to nearly double that of controls in tamoxifen-treated cells.

We also investigated the effects of hormone treatment on the expression of VEGFR-2 in MCF-7 cells. Endogenous levels of VEGFR-2 were not detectable in MCF-7 cells incubated in hormone-free medium or with addition of oestradiol, tamoxifen, or oestradiol+tamoxifen.

VEGF is known to exert its effects by acting on endothelial cells primarily via VEGFR-2. We therefore set up a series of experiments to investigate the effects of oestradiol and tamoxifen on HUVEC. In the first set of experiments we treated HUVEC with oestradiol, tamoxifen, or a combination of the two and measured cellular expression of VEGFR-2. We detected low levels of VEGFR-2 in HUVEC and found no difference in expression levels in hormone-treated cells compared to controls (mean 13±1.9 pg mg^−1^ protein for all treatment groups).

**Figure 2:**
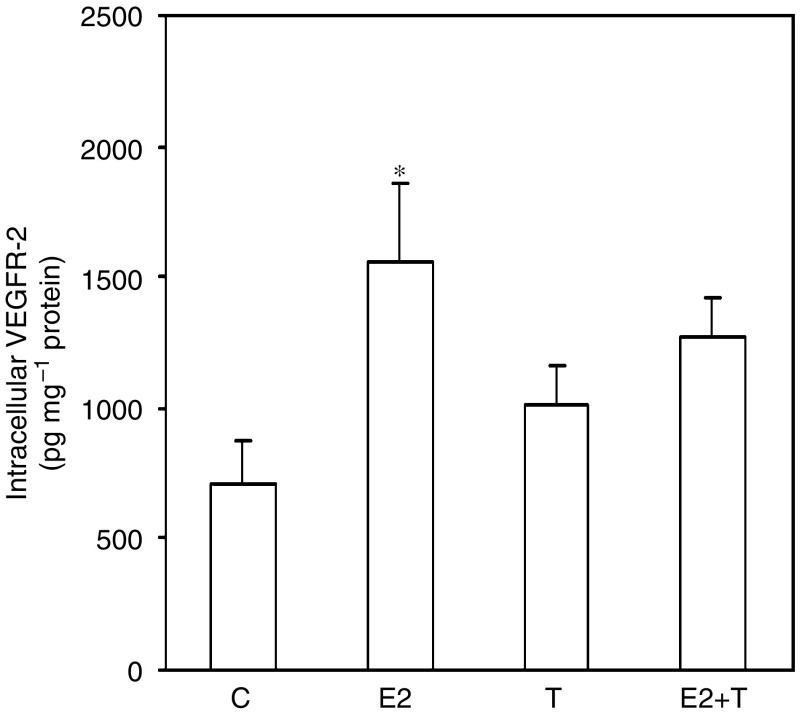
Cell-associated VEGFR-2 after exposure of HUVEC to hormone-treated MCF-7 cell supernatants in culture. MCF-7 cells were treated as described in Figure 1. After 24 h, the MCF-7 cell supernatants (C, E2, T, and E2+T groups, respectively) were transferred to untreated HUVEC and thereafter changed every day. VEGFR-2 was measured in HUVEC cell lysates using ELISA after 3 days in culture (*n*=4–5 in each group, two separate experiments; ^*^P<0.05 as compared with control cells).

In a tumour, endothelial cells are exposed to and affected by products secreted by cancer cells. We therefore set up a second set of experiments and treated HUVEC with cell culture media collected from hormone-treated MCF-7 cells. We found that VEGFR-2 was upregulated in all groups exposed to conditioned media from MCF-7 cells compared to hormone-treated HUVEC. Human umbilical vein endothelial cells exposed to conditioned media from oestradiol-stimulated MCF-7 cells exhibited significantly higher levels of VEGFR-2 as compared to control cells incubated with media from nonhormone treated MCF-7 cells (*P*<0.05; Figure 2).

Low levels of extracellular VEGF were detected in HUVEC supernatants with no differences observed after addition of hormones (mean 0.94±0.27 pg mg^−1^ protein for all treatment groups). No significant differences were detected in the secretion of VEGF by HUVEC following incubation with conditioned media from hormone-treated MCF-7 cells (data not shown).

**Figure 3:**
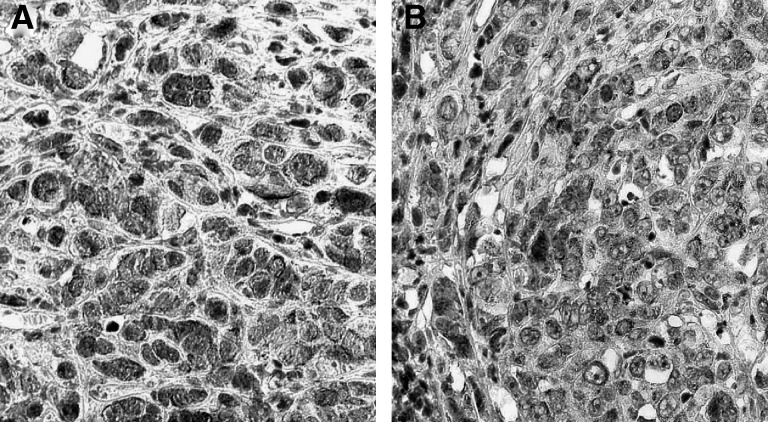
Immunohistochemistry of VEGFR-1 in tumour sections. Solid MCF-7 tumours were established in nude mice as described in the Materials and Methods section. No differences were observed in the intensity of staining in tumours from the two treatment groups, oestradiol (E2) and oestradiol+tamoxifen (E2+T). Representative sections from tumours are shown. (**A**) Tumour section from an oestradiol-treated tumour. (**B**) Tumour section from an oestradiol+tamoxifen-treated tumour.

Our *in vitro* results revealing higher levels of cell-associated sVEGFR-1/VEGFR-1 in oestradiol+tamoxifen treated cells compared with oestradiol alone prompted further investigation into the effects of oestradiol and tamoxifen on intratumoral levels of sVEGFR-1/VEGFR-1 *in vivo*. Immunohistological staining with anti-VEGFR-1 was performed on tumour sections from solid MCF-7 tumours. All treatment groups exhibited similar intracellular localisation of sVEGFR-1/VEGFR-1. No differences were detected between the groups. Representative tumour sections are shown in Figure 3A and B.

**Figure 4:**
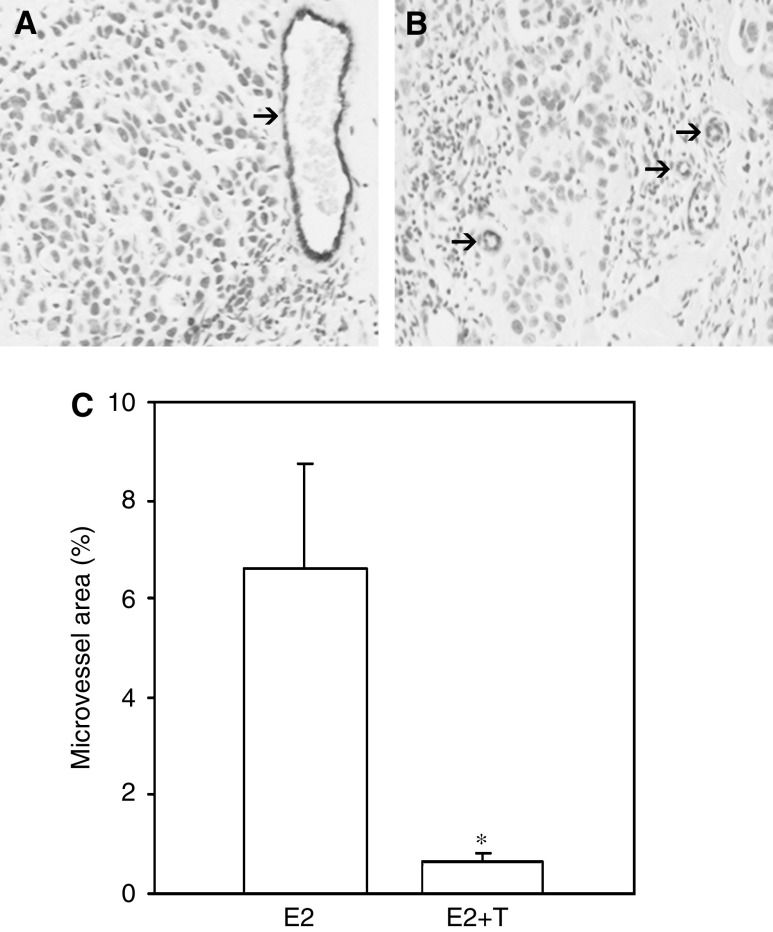
Tumour vasculature in solid MCF-7 tumours in nude mice. Mice were treated as described in Figure 3 and tumour sections were stained with anti-von Willebrand's factor. Representative tumour sections from the oestradiol and the oestradiol+tamoxifen-treated groups are shown in (**A**) and (**B**), respectively. Examples of positively stained vessels are marked with arrows. (**C**) Tumour vessel area based on positive tumour staining for von Willebrand's factor and conducted in a blinded manner. Vessel area in percent was measured from three hot-spots from three different tumours in each group (^*^P<0.05).

To evaluate the biological relevance of the balance between sVEGFR-1 and VEGF on tumour vasculature *in vivo*, we quantified vessel area in size-matched tumours from hormone-treated nude mice. Using immunohistological staining with anti-von Willebrand's factor and quantifying vessel area in a blinded manner, we observed significantly decreased vessel area on tumour sections from animals treated with a combination of oestradiol+tamoxifen compared to oestradiol treatment only (*P*<0.05; Figure 4C). Representative tumour sections are shown in Figure 4A and B. Microscopic sections of the tumours did not reveal any necrotic areas in either of the treatment groups. Following 2 weeks of treatment, tumours in the oestradiol+tamoxifen group were significantly smaller than those in the oestradiol group (566±68 mm^3^ compared to 1266±291 mm^3^; *P*<0.05). No mice showed signs of weight loss or deteriorated general condition.

## DISCUSSION

In this study we show in breast cancer cells *in vitro* that oestradiol decreased extracellular sVEGFR-1 and increased extracellular VEGF *in vitro*. The addition of tamoxifen significantly reversed these effects. The extracellular ratio of sVEGFR-1/VEGF was nine times higher following tamoxifen treatment alone compared to control cells. The ratio in oestradiol-treated cells was approximately one-third that of control cells. Moreover, in HUVEC, incubation with supernatants from oestradiol-treated MCF-7 cells resulted in significantly higher cell-associated VEGFR-2 levels as compared to control cells.

Tamoxifen has been shown to have an antiangiogenic effect on breast cancer, but the mechanisms of this effect have not been fully explored. We propose that the antiangiogenic effect of tamoxifen could be explained, at least in part, by its effects on VEGF and sVEGFR-1 as shown in the present study. Oestradiol, on the other hand, has been shown to exert proangiogenic effects in breast cancer, mediated in part by its potent effects on VEGF (Dabrosin *et al*, 2003a, 2003b). The present study suggests that oestrogen influences both sVEGFR-1 levels secreted from cancer cells and VEGFR-2 expression on endothelial cells, resulting in a net proangiogenic effect. The *in vitro* data were confirmed by the antiangiogenic effect of tamoxifen observed in solid tumours *in vivo*. Our results are in agreement with a recent study describing decreased sVEGFR-1 expression in oestrogen-receptor positive cell lines following treatment with oestradiol (Elkin *et al*, 2004). In this study, the oestrogen-mediated decrease in sVEGFR-1 expression *in vitro* was accompanied by a significant increase in angiogenesis *in vivo* (Elkin *et al*, 2004).

Although differences in cell-associated sVEGFR-1/VEGFR-1 were observed *in vitro* in our present study, immunohistological staining of sVEGFR-1/VEGFR-1 in tumour sections did not reveal any significant differences between oestradiol and oestradiol+tamoxifen-treated animals. The intracellular staining observed is consistent with previous findings, suggesting that MCF-7 cells express only sVEGFR-1 and virtually no cell-membrane bound VEGFR-1 (Elkin *et al*, 2004). sVEGFR-1 diffuses freely into the extracellular space where it is suggested to be biologically active (Hornig and Weich, 1999), and it is uncertain if soluble extracellular proteins can be detected by immunohistochemical techniques (Zhang *et al*, 2000). It is possible that immunohistochemistry, considered a semiquantitative method, is not sensitive enough to detect intracellular differences between treatment groups. A direct measurement of sVEGFR-1 *in situ* in the tumour would be a preferable way to investigate sVEGFR-1. However, due to sVEGFR-1's large molecular size, the technique of microdialysis, which we have used in previous studies (Dabrosin *et al*, 2003a; Garvin and Dabrosin, 2003), could not be employed to measure the *in vivo* levels of sVEGFR-1 in solid tumours.

In this study, we also show that incubation of HUVEC with supernatants from oestradiol-treated MCF-7 cells resulted in increased cell-associated VEGFR-2 levels as compared to control cells. When HUVEC were cultured in regular growth media with and without hormones added VEGFR-2 was detected at very low levels. However, when HUVEC were cultured in supernatants from MCF-7 cells, VEGFR-2 was upregulated in all groups with a significantly higher level in HUVEC grown in media from oestrogen-treated cancer cells compared with cancer cell media without hormones added. It has been demonstrated that VEGF upregulates the expression of VEGFR-2 in endothelial cells (Murata *et al*, 2000). Our results suggest that the excess of unbound VEGF detected in oestrogen-treated MCF-7 cell supernatants may contribute to increased expression of VEGFR-2 in HUVEC. However, the influence of other hormone responsive factors secreted from hormone-stimulated MCF-7 cells may also contribute to higher VEGFR-2 levels. In addition, it has been demonstrated that HUVEC express oestrogen receptors (Kim-Schulze *et al*, 1996; Venkov *et al*, 1996), thus direct hormonal effects on HUVEC cannot be excluded although not apparent in our results. It is unlikely that HUVEC-derived VEGF contributes to this upregulation, as very low levels of extracellular VEGF were detected in HUVEC supernatants. Moreover, VEGF secretion by HUVEC did not appear to be affected by direct hormone treatment or by incubation with supernatants from hormone-treated MCF-7 cells.

VEGFR-2 was not detected in MCF-7 cells irregardless of the absence or presence of hormones. Although it has been proposed that VEGFR-2 expression in breast cancer cells may function as an autocrine loop in which VEGF regulates growth of cancer cells in an animal model for breast cancer (Xie *et al*, 1999), this does not appear to be the case for MCF-7.

In summary, we show that tamoxifen and oestradiol exert dual effects on the angiogenic environment in breast cancer by regulating extracellular levels of important angiogenic factors secreted by cancer cells as well as by exerting effects on receptor expression of endothelial cells. Our results suggest an oestrogen-driven angiogenic switch, explained by the inhibition of sVEGFR-1 and stimulation of VEGF and VEGFR-2, tipping the angiogenic scale to favour angiogenesis, and possibly contributing to breast carcinoma progression. Tamoxifen, on the other hand, tips the balance to favor angiogenic inhibition. We emphasise the importance of investigating the regulation of proteins where they are biologically active, in the extracellular space in the case of VEGF and sVEGFR-1. Moreover, the results of this study illustrate the importance of the interaction between tumour cells and local microenvironment in regulating angiogenesis and the critical role of sex steroids in this process.
